# Dynamic Sweep Experiments on a Heterogeneous Phase Composite System Based on Branched-Preformed Particle Gel in High Water-Cut Reservoirs after Polymer Flooding

**DOI:** 10.3390/gels9050364

**Published:** 2023-04-25

**Authors:** Xianmin Zhang, Yiming Zhang, Haicheng Liu, Shanshan Li, Lijie Liu

**Affiliations:** 1 Key Laboratory of Unconventional Oil & Gas Development, Ministry of Education, China University of Petroleum (East China), Qingdao 266580, China; 2 School of Petroleum Engineering, China University of Petroleum (East China), Qingdao 266580, China; 3 Research Institute of Exploration and Development, Shengli Oilfield Company, SINOPEC, Dongying 257015, China

**Keywords:** polymer flooding, heterogeneous phase composite system, branched-preformed particle gel, image processing, sweep efficiency

## Abstract

Heterogeneous phase composite (HPC) flooding technology that is based on branched-preformed particle gel (B-PPG) is an important technology for enhancing oil recovery in high water-cut reservoirs. In this paper, we conducted a series of visualization experiments under the condition of developed high-permeability channels after polymer flooding, with respect to well pattern densification and adjustment, and HPC flooding and its synergistic regulation. The experiments show that for polymer-flooded reservoirs, HPC flooding can significantly reduce the water cut and increase oil recovery, but that the injected HPC system mainly advances along the high-permeability channel with limited sweep expansion. Furthermore, well pattern densification and adjustment can divert the original mainstream direction, which has a positive effect on HPC flooding, and can effectively expand the sweeping range under the synergistic effect of residual polymers. Due to the synergistic effect of multiple chemical agents in the HPC system, after well pattern densification and adjustment, the production time for HPC flooding with the water cut lower than 95% was significantly prolonged. In addition, conversion schemes, in which the original production well is converted into the injection well, are better than non-conversion schemes in terms of expanding sweep efficiency and enhancing oil recovery. Therefore, for well groups with obvious high-water-consuming channels after polymer flooding, the implementation of HPC flooding can be combined with well pattern conversion and intensification in order to further improve oil displacement.

## 1. Introduction

The mature waterflooding oilfields in eastern China are mainly terrestrial sedimentary sandstone reservoirs with complex structures and serious heterogeneity [1,2]. After decades of waterflooding development, the water injectivity of each layer varies greatly, resulting in small swept volumes, and the injected water often advances along the high-permeability layers with a high degree of water flooding and low movable oil saturation. The remaining oil distribution is highly dispersed and locally concentrated [3,4].

Polymer flooding technology has become an important means of enhancing oil recovery in China’s high water-cut oilfields and has been applied in large-scale industrial applications in the Shengli and Daqing oilfields [5,6]. Studies have shown that polymer flooding can effectively expand the swept volume and improve displacement efficiency. After polymer flooding, the final oil recovery can generally reach 40–50%, an increase of 7–15% compared with water flooding [7]. However, approximately 50% of the crude oil still remains underground, representing a significant opportunity to further improve oil recovery [8,9,10]. After polymer flooding, the reservoir heterogeneity is generally further intensified, and the distribution of the remaining oil is more scattered. Subsequently, injected fluids easily form ineffective channeling along dominant zones, which seriously affects the expansion of the swept volume. In addition, the residual polymer can become stuck in the low-permeability zone, which hinders contact between the subsequent oil displacement agents and the crude oil surface, leading to the “secondary sweep problem” [11,12]. This poses a great challenge to the deep development of reservoirs after polymer flooding.

Many scholars have investigated methods for further improving oil recovery after polymer flooding, including through foam flooding [13,14], alkali–surfactant–polymer (ASP) flooding [15], gel treatment [16], nanoparticle treatment [17], etc.; nevertheless, these technologies present defects, such as being easily flushed by injected water, and poor stability. Heterogeneous phase composite (HPC) flooding technology that is based on branched-preformed particle gel (B-PPG) is an important technology for enhancing oil recovery in ultra-high water-cut reservoirs, and has been shown to effectively increase oil recovery and prolong production time [18,19,20]. B-PPG is obtained by the cross-linking reaction of the main chemical agent, which cross-links the agent and initiator under certain conditions, followed by processing after drying, crushing and sieving. The particles have strong viscoelasticity, good suspension and deformability, and can maintain stability in most reservoirs and various water formations. [21,22]. Research has shown that the main mechanism of B-PPG is to block cracks, leading to water diversion [23,24]. When injected into the formation, the water-swelling particles can pass through pore throats of a smaller diameter than themselves, and block high-permeability channels, thereby expanding the subsequent water-sweeping area [25,26,27]. In order to better employ the plugging effect of B-PPG, Cui [28] designed a heterogeneous phase composite flooding (HPCF) system that was composed of a polymer, surfactant and B-PPG. Experiments have shown that compared with polymer flooding, HPFC can further expand the swept range and improve oil recovery.

Well pattern adjustment is another conventional method of oil production in unswept areas [29,30,31,32]. This method can effectively improve flow field distribution by infilling and transferring wells, that is, to adjust the original injection–production well pattern by infilling injection–production wells locally or by transferring production wells into injection in areas with an imperfect injection–production relationship, thereby producing more remaining oil in areas that are not easily swept by the HPCF system; this significantly reduces the underground oil saturation of reservoirs [33].

Since the distribution of the remaining oil changes greatly after polymer flooding and the pore structure is more complex [34], it is difficult to meet the demand for further the significant enhancement of oil recovery. In recent years, many scholars have studied technologies to further enhance oil recovery after polymer flooding. Gao et al. evaluated the synergistic effect of PPG and ASP after polymer flooding using micro-displacement experiments [35]; they concluded that the synergy of PPG and ASP can not only further improve oil recovery, but also reduce polymer consumption and improve economic benefits. Gong et al. investigated a combined B-PPG and hydrolyzed polyacrylamide (HPAM) system; they found that the synergistic displacement effect was better than that of any single agent and further enhanced oil recovery after polymer flooding [36]. Li et al. performed core displacement experiments on an enhanced foam flooding system that was composed of a polymer, foam agent and nitrogen after polymer flooding [37,38]; they confirmed that the system could effectively improve reservoir stability, and that the improvement in the injection profiles could significantly enhance oil recovery. Although these methods significantly improved the oil displacement efficiency, they are still very limited in producing remaining oil in unswept areas. Therefore, research on a combination of the HPC system and a well pattern adjustment (WA) has attracted extensive attention. Sun et al. studied the synergistic effect of streamline adjustment and HPC flooding after polymer flooding [39,40]. This method adjusts the well pattern after polymer flooding to produce crude oil in the unswept areas and increase the action range of the HPC system, thereby significantly expanding the swept area and enhancing oil recovery. After synergistic application, the mainstream direction changes and the injection pressure also increases, which leads to an obvious reduction in the water cut and enhancement in the oil recovery.

However, the existing HPC systems are rarely applied in the field, and their displacement evaluation is limited. It is, therefore, necessary to focus on the characteristics of highly developed dominant channels after polymer flooding, and further study the effect of well pattern adjustment and HPC flooding on the dynamic sweep regulation, as well as the synergistic mechanism that is involved in expanding the sweep with the residual polymer.

In this paper, we report on an HPC flooding pilot test block after polymer flooding and well pattern adjustment, performed based on the characteristics of the Shengli Oilfield in China. We conduct a series of visualization experiments after polymer flooding using a two-dimensional sand pack visual displacement device. We investigate the dynamic sweep characteristics of well pattern densification and adjustment, HPC flooding and their synergistic regulation under the condition of developed high-permeability channels. In addition, we explain the influence of dominant high-permeability channels, residual polymers and well pattern adjustment after polymer flooding on the dynamic sweep of HPC flooding, and provide theoretical guidance for the large-scale and beneficial development of similar reservoirs after polymer flooding.

## 2. Results and Discussion

### 2.1. Dynamic Sweep Evaluation in HPCF Experiments

**Figure 1:**
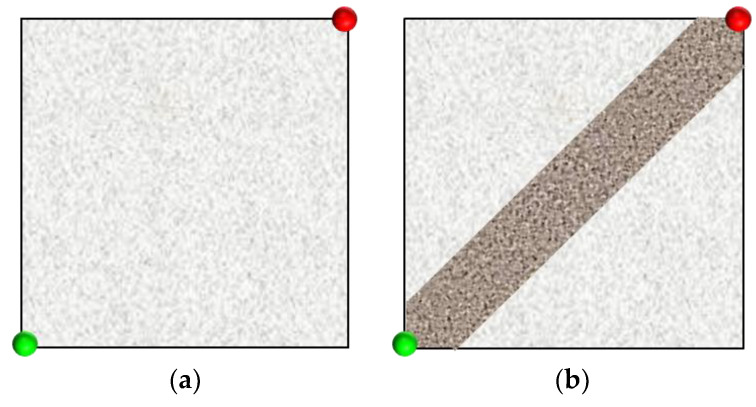
Schematic diagram of sand pack models of different heterogeneities. (**a**) Weakly heterogeneous model with 34.8% porosity. (**b**) Strongly heterogeneous model with 33.5% porosity. The green and red circles, respectively, represent injection wells and production wells. The diagonal stripe represents the high-permeability zone.

In our experiments, we designed a five-point well pattern unit, consisting of one injection well and one production well; this was based on the actual well pattern characteristics of polymer flooding in the Shengli Oilfield [18]. The five-point well pattern unit was then used to design strongly heterogeneous and weakly heterogeneous models (Figure 1). In this figure, the green circle represents the injection well, and the red circle represents the production well. The weakly heterogeneous model was filled with glass microspheres of an average size of 60 mesh, while the strongly heterogeneous model was filled with glass microspheres of an average size of 40 mesh in the high-permeability zone and 80 mesh in the low-permeability zone. The average porosities of these two models were 33.5% and 34.8%, respectively.

#### 2.1.1. Visual Experimental Results

**Figure 2:**
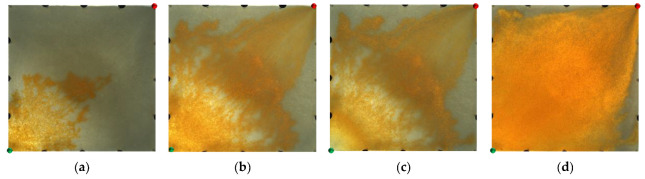
Water flooding and polymer flooding processes in weakly heterogeneous model. (**a**) Initial stage of water flooding. (**b**) End stage of water flooding. (**c**) Initial stage of polymer flooding. (**d**) End stage of polymer flooding.

From the displacement processes in Figure 2, it can be seen that in the weakly heterogeneous model, the injected water displacement was relatively balanced in the water flooding stage, and there were some unswept areas at the edge of the model. In the polymer flooding stage, after the injection of the polymer solution, the viscosity of the injected water increased and the water–oil mobility ratio improved. Coupled with its viscoelastic effect, this dragged the blind terminal and droplet residual oil at the edge of the pores along, thereby forming a stable oil channel. In the area swept by the polymer front, the displaced residual oil was enriched with time. The advance of the polymer front was relatively balanced, and there were few unswept areas between the injection and production wells.

**Figure 3:**
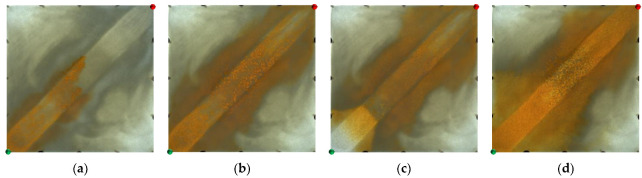
Water flooding and polymer flooding processes in strongly heterogeneous model. (**a**) Initial stage of water flooding. (**b**) End stage of water flooding. (**c**) Initial stage of polymer flooding. (**d**) End stage of polymer flooding.

However, for the strongly heterogeneous model, it can be seen from Figure 3 that in the water flooding stage, the injected water was mainly displaced along the high-permeability channel and had a very limited dynamic sweeping area. In the polymer flooding stage, the massive polymer aggregation increased the seepage resistance of the high-permeability channel, which expanded the sweeping range of the subsequent injected water and achieved balanced water flooding; however, there was still a large amount of oil remaining in the marginal area. The sweep expansion effect of the strongly heterogeneous model was worse than that of the weakly heterogeneous model.

**Figure 4:**
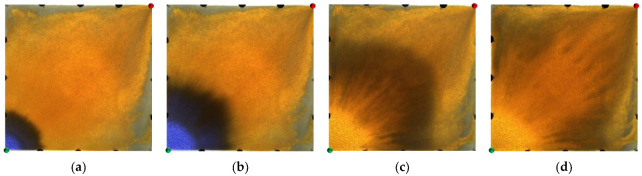
Heterogeneous phase composite (HPC) flooding processes in the weakly heterogeneous model. (**a**) Initial stage of HPC injection. (**b**) End stage of HPC injection. (**c**) Initial stage of water injection. (**d**) Water cut stage of 95%.

**Figure 5:**
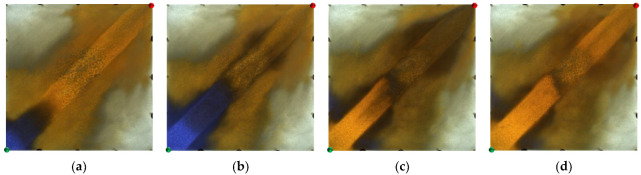
HPC flooding processes in strongly heterogeneous model. (**a**) Initial stage of HPC injection. (**b**) End stage of HPC injection. (**c**) Initial stage of water injection. (**d**) Water cut stage of 95%.

As shown in Figure 4, in the weakly heterogeneous model, after polymer flooding, the injected HPC system blocked the pore throats, and changed the direction of the subsequently injected water, thus expanding the sweep efficiency and effectively utilizing the residual oil in the unswept area. In contrast, in the strongly heterogeneous model, it can be seen from Figure 5 that the HPC system established a good frictional resistance along the high-permeability channel, which forced more injected water to divert and to spread into the marginal low-permeability zone.

#### 2.1.2. Evaluation of Sweep Efficiency

**Figure 6:**
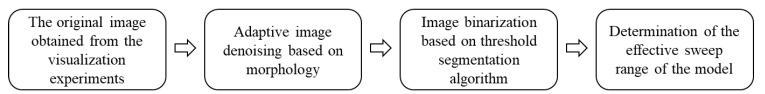
Image processing technology flowchart.

**Figure 7:**
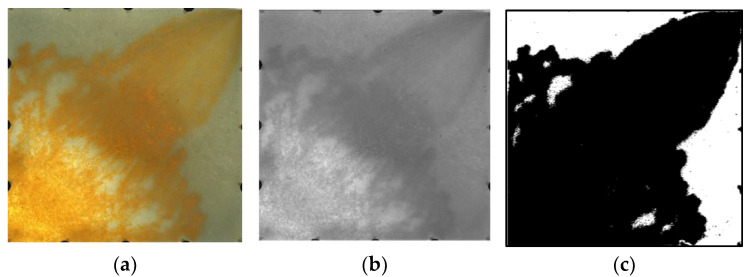
Experimental image processing technology. (**a**) Original image. (**b**) Grayscale image. (**c**) Threshold segmentation image.

The sweep efficiency is a very important factor affecting reservoir recovery [41] and can intuitively reflect the sweep degree of the displacement phase. At present, the determination methods for sweep efficiency include the core test method and the numerical simulation method. Of these, the core test method cannot intuitively display the swept position of the displacement phase, while the numerical simulation method cannot accurately describe the swept range for the actual model. In addition to these two methods, image processing technology for a visual model has also been applied for the determination of sweep efficiency [42]. Compared with traditional methods, the image processing method can divide the swept areas conveniently and intuitively. A flowchart showing the image processing technology is provided in Figure 6. By graying the obtained images [43,44,45] and then thresholding the images according to the two-dimensional Otsu image threshold segmentation algorithm [46], an appropriate grayscale image threshold was selected; then, the target and background were segmented via the grayscale threshold (Figure 7) so as to convert the grayscale image into a binary image that could reflect its local and overall characteristics.

**Figure 8:**
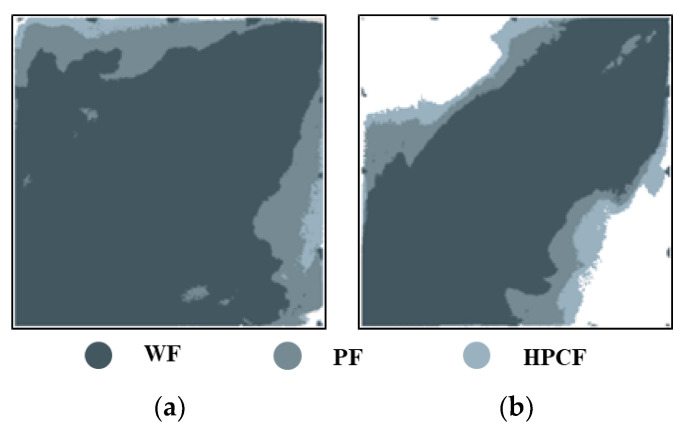
Stacked diagrams of swept range of different models (WF (water flooding), PF (polymer flooding) and HPCF (HPC flooding) from deep to shallow). (**a**) Weakly heterogeneous model. (**b**) Strongly heterogeneous model.

Figure 8 shows the stacked diagrams of the sweep efficiency that were obtained by processing the images and then using MATLAB to determine the effective sweep range of the model. It can be seen that the HPC system can effectively improve sweep efficiency. Due to the good viscoelasticity and plugging properties of the HPC system, the subsequent injected water diverted more to both sides of the mainstream area, thereby completely sweeping the weakly heterogeneous model, and significantly improving the swept range of the strongly heterogeneous model. The sweep efficiencies of the weakly and strongly heterogeneous models after HPC flooding were 98.45% and 79.87%, respectively, and compared with those after polymer flooding, increased by 5.93% and 8.99%, respectively.

#### 2.1.3. Water Cut and Recovery

**Figure 9:**
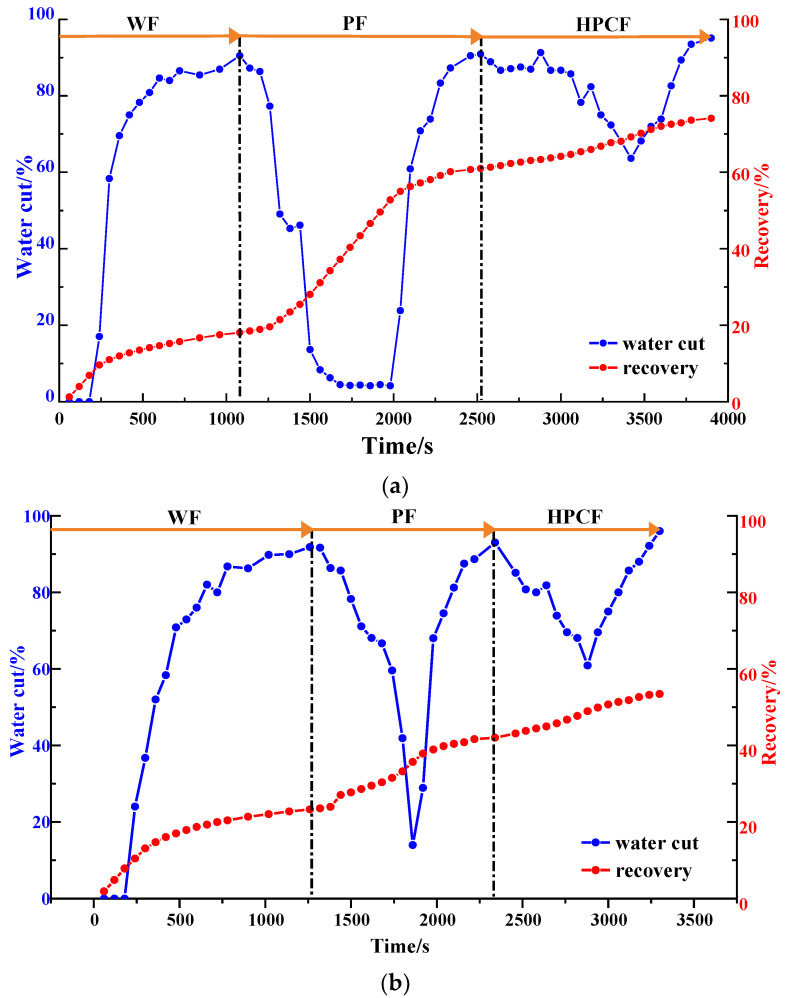
Diagrams of water cut and recovery in heterogeneous models. (**a**) Weakly heterogeneous model. (**b**) Strongly heterogeneous model.

Via an analysis of the water cut and the recovery factor of the different heterogeneous models (Figure 9 and Table 1), it can be seen that the water cut decreased significantly in the polymer flooding stage, and the recovery factor increased significantly. In the strongly heterogeneous model, the high-permeability channel produced less excess oil in each displacement stage, which limited the enhancement of the recovery factor and the extension of the recovery time. The recovery factors for the weakly and strongly heterogeneous models after polymer flooding were 60.13% and 42.02%, respectively. The HPC system significantly reduced the water cut and enhanced the oil recovery. The water cut for the two models dropped to 63.64% and 60.87%, respectively, and compared with those after polymer flooding, their recovery factors after HPC flooding increased by 13.97% and 11.43%, respectively. However, the increase in the sweep efficiency was limited, ranging from 5.93% to 8.99%. Especially for the strongly heterogeneous model, the sweep efficiency was only 79.87% after HPC flooding, meaning that there is still huge potential to further improve the oil recovery.

### 2.2. Dynamic Sweep Evaluation in WAF Experiments

**Figure 10:**
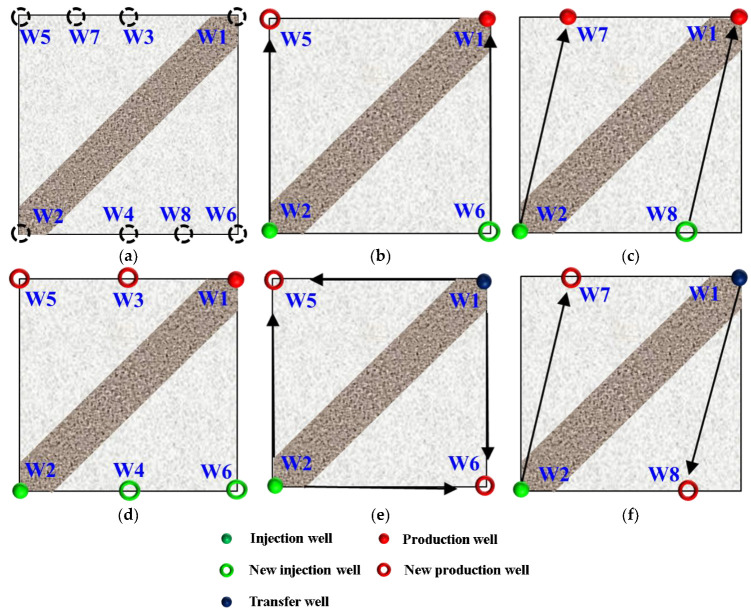
Well pattern densification adjustment schemes. (**a**) Original well location. (**b**) Scheme 1. (**c**) Scheme 2. (**d**) Scheme 3. (**e**) Scheme 4. (**f**) Scheme 5. Arrow represents flooding direction.

The efficient development of unswept remaining oil is the key to improving oil recovery [39]. After HPC flooding, due to the high-permeability channel between the injection and production wells, there was still a large amount of oil remaining in the non-mainstream areas, with a limited increase in the sweep efficiency, especially for the strongly heterogeneous model. Well pattern densification and adjustment (WA) is an effective measure by which to change the flow field direction, forcibly utilize the remaining oil and expand the sweep efficiency. In order to study the influence of different WA schemes on the oil displacement effect after polymer flooding, five post-polymer flooding schemes were designed for the strongly heterogeneous model (Figure 10). The five schemes were divided into non-conversion schemes, Schemes 1, 2 and 3, and conversion schemes, Schemes 4 and 5, with the original production wells, W1 in Schemes 4 and 5, being converted into injection wells.

#### 2.2.1. Visual Experimental Results

**Figure 11:**
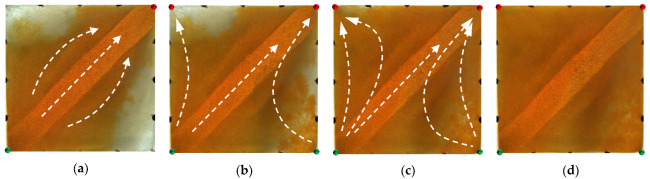
Scheme 1 in WAF experiments. (**a**) Initial stage of WAF. (**b**) Water cut stage of 50%. (**c**) Water cut stage of 80%. (**d**) Water cut stage of 95%. White arrows represent the flooding direction.

**Figure 12:**
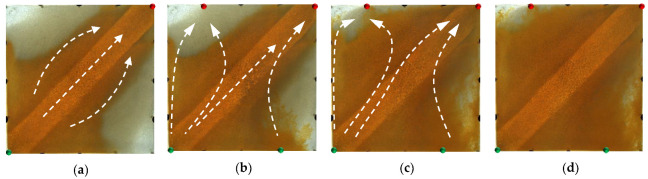
Scheme 2 in WAF experiments. (**a**) Initial stage of WAF. (**b**) Water cut stage of 50%. (**c**) Water cut stage of 80%. (**d**) Water cut stage of 95%. White arrows represent the flooding direction.

**Figure 13:**
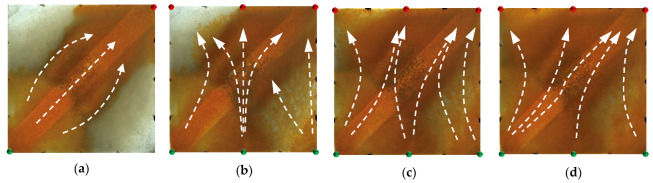
Scheme 3 in WAF experiments. (**a**) Initial stage of WAF. (**b**) Water cut stage of 50%. (**c**) Water cut stage of 80%. (**d**) Water cut stage of 95%. White arrows represent the flooding direction.

For the non-conversion schemes, as shown in Figure 11, Figure 12 and Figure 13, after the well pattern adjustment, most of the injected water from the original injection well still flushed along the high-permeability channel, and under the action of the residual polymer, the water injected from the new well effectively expanded the sweep efficiency. However, after the injected water from the new injection well pushed the residual polymer into the high- permeability channel, its seepage resistance increased, and due to the diversion effect between the injection wells, more injected water was diverted in the direction of the new production wells. Under the synergistic effect of the residual polymer, the sweeping range effectively expanded and the balanced displacement of the remaining oil was realized.

**Figure 14:**
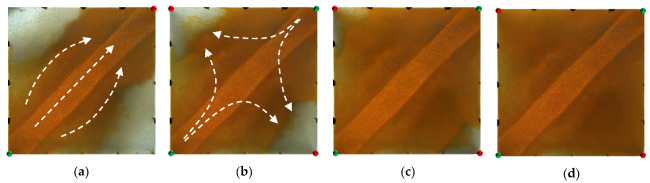
Scheme 4 in WAF experiments. (**a**) Initial stage of WAF. (**b**) Water cut stage of 0%. (**c**) Water cut stage of 80%. (**d**) Water cut stage of 95%. White arrows represent the flooding direction.

**Figure 15:**
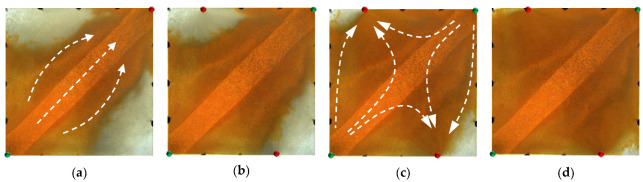
Scheme 5 in WAF experiments. (**a**) Initial stage of WAF. (**b**) Water cut stage of 0%. (**c**) Water cut stage of 80%. (**d**) Water cut stage of 95%. White arrows represent the flooding direction.

Figure 14 and Figure 15 show the experimental results of the two conversion schemes (Schemes 4 and 5). After infilling two production wells, the direction of the original mainstream was disrupted, and the original non-mainstream area became the mainstream area. Injected water preferentially displaced through the high-permeability channel and then diverted due to the diversion effect between the injection wells. Since a large amount of polymer remained near the high-permeability zone after polymer flooding, the diversion of the injected water to the oil well expanded the action range of the residual polymer, resulting in a more balanced displacement effect. After the injected water broke through, the formation of the mainstream displacement path effectively expanded the swept range of water flooding, and the remaining oil in the unswept areas was effectively produced. These five WAF schemes could effectively utilize the remaining oil in the unswept areas, and the conversion of the production well could change the original mainstream direction.

#### 2.2.2. Quantitative Results Analysis

**Figure 16:**
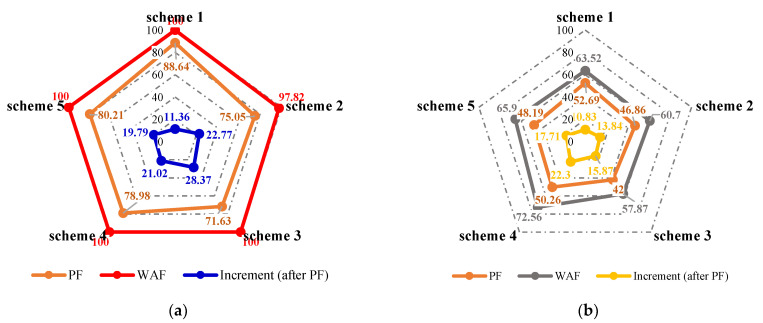
Sweep efficiency and recovery in WAF experiments. (**a**) Sweep efficiency. (**b**) Recovery.

The changes in the sweep efficiency at different flooding stages are shown in Figure 16a. After polymer flooding, the well pattern adjustment could effectively utilize the remaining oil in the unswept areas. For the non-conversion schemes, since the original injection and production wells had not changed, the injected water from the original or new injection well preferentially flushed along or toward the high-permeability channel, and under the effect of the residual polymer and diversion line, the swept area of injected water further expanded. The larger the flow field transition angle after well pattern adjustment, the higher the sweep efficiency. After the well pattern adjustment, the flow field transition angle in Scheme 2 was obviously the smallest, as the sweep efficiency was 97.82%. For the conversion schemes, after converting the original production well to the injection well and infilling another two new production wells, the mainstream direction of the original flow field was broken, and the original non-mainstream area became the mainstream area, which effectively expanded the sweeping range of the injected water. The larger the flow field transition angle after the well pattern adjustment was, the better the expanded sweep of water flooding was.

**Figure 17:**
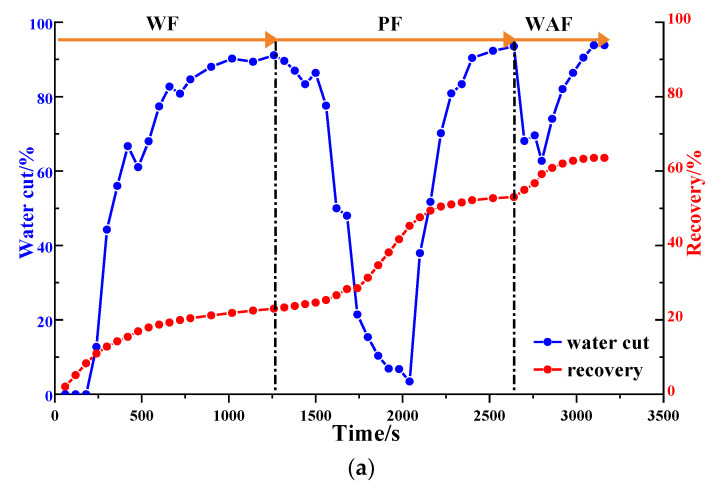

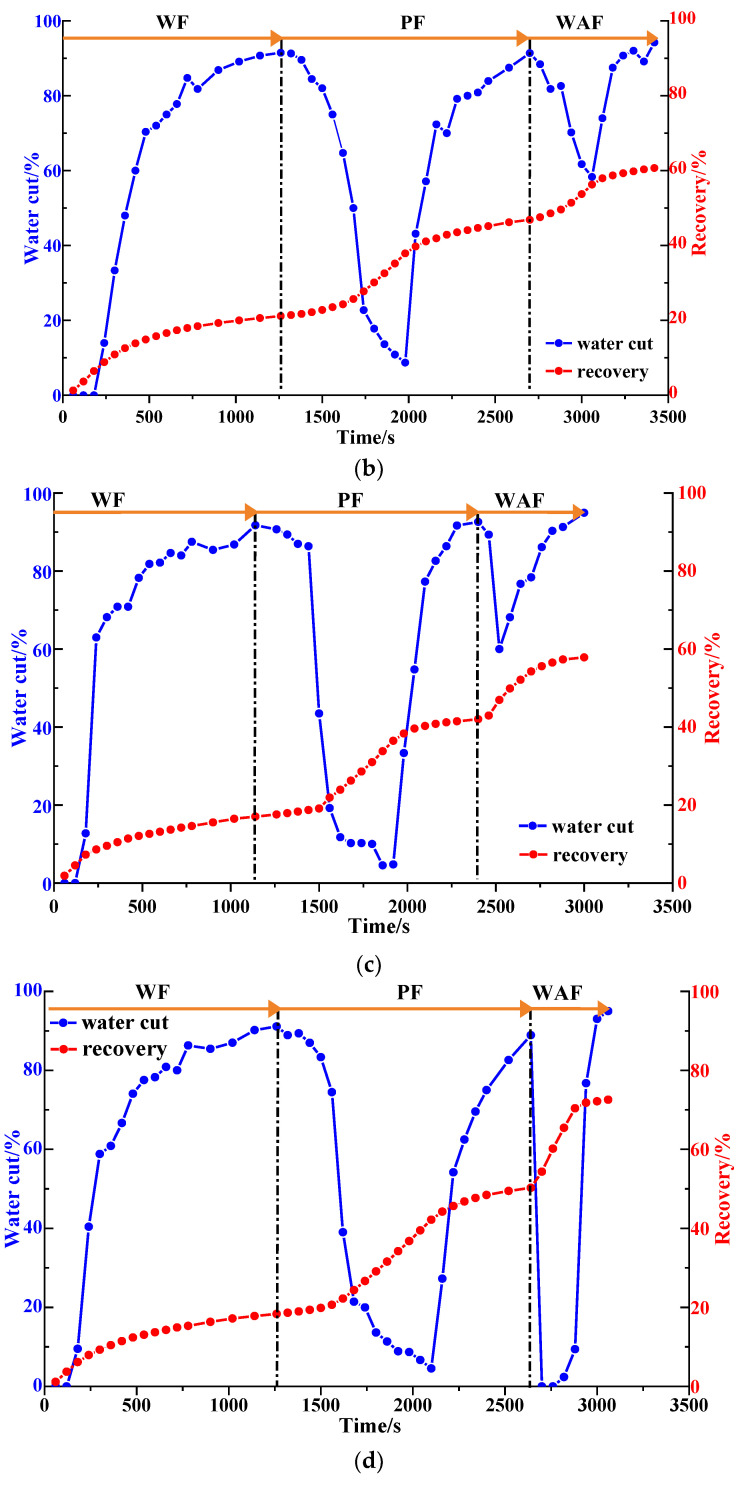

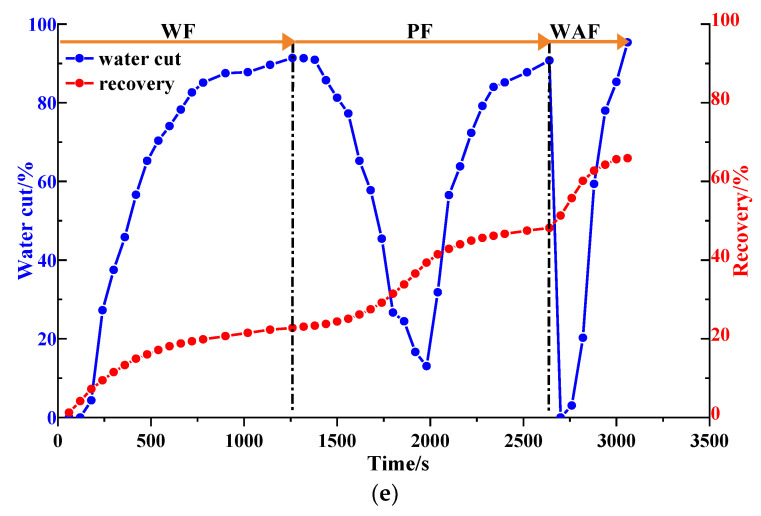
Water cut and recovery change results for five WAF experiments. (**a**) Scheme 1. (**b**) Scheme 2. (**c**) Scheme 3. (**d**) Scheme 4. (**e**) Scheme 5.

As shown in Figure 16b and Figure 17, after well pattern adjustment, the recovery factor for each scheme improved to a certain extent. In the non-conversion schemes, due to the ineffective water displacement along the high-permeability channel, although the water cut decreased after the well pattern adjustment, the decrease was so limited as to result in a small increase in the recovery, as shown in Figure 17a–c. Among the non-conversion schemes, Scheme 3 had the highest enhanced oil recovery (EOR) value at 15.87%, followed by Scheme 2 with an EOR value of 13.84%, and Scheme 1 with the lowest EOR value at 10.83%. However, for the conversion schemes, the mainstream area significantly increased after the transfer injection, and under the synergistic effect of residual polymers, the water cut decreased significantly compared with those non-conversion schemes, as shown in Figure 17d,e. There was a certain period of anhydrous or extremely low water cut production in both schemes, and their recoveries significantly improved. As new seepage channels were formed after the injected water broke through, the water cut increased rapidly, and the development characteristics of the high-water-consumption zones became obvious. After well pattern adjustment, the conversion schemes improved the oil recovery significantly better than the non-conversion schemes. The recovery factors of the two conversion schemes after polymer flooding increased by 22.3% and 17.71%, respectively. Furthermore, after polymer flooding, the effective production time in all five WAF experiments was shorter than that in the HPCF experiment (960 s, as shown in Figure 9b), ranging from 420 s to 720 s.

### 2.3. Dynamic Sweep Evaluation in WAHPCF Experiments

Although the sweep efficiency increased after well pattern densification and adjustment, after the injected water broke through, the water cut rose rapidly, seriously affecting the effective production time. After water flooding, the oil saturation in the original unswept area was still high, and the displacement efficiency was low [36]. According to previous experiments [39], the HPC system effectively blocked the dominant channels, expanding the water flooding range, and the well pattern densification and adjustment expanded the scope of the HPC system, which played a guiding role in the HPC system. In order to study the influence of the HPC system under different well patterns, five WAHPCF schemes were designed on the basis of well pattern densification and adjustment.

#### 2.3.1. Visual Experimental Results

**Figure 18:**
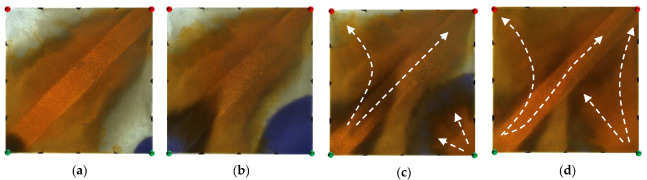
Scheme 1 in WAHPCF experiments. (**a**) Initial stage of HPC injection. (**b**) End stage of HPC injection. (**c**) Initial stage of water injection. (**d**) Water cut stage of 95%. White arrows represent the flooding direction.

**Figure 19:**
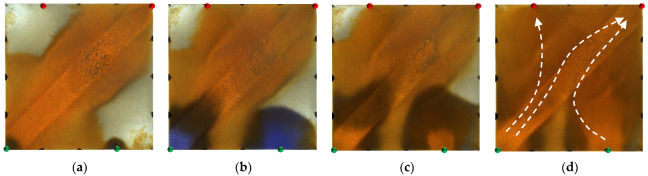
Scheme 2 in WAHPCF experiments. (**a**) Initial stage of HPC injection. (**b**) End stage of HPC injection. (**c**) Initial stage of water injection. (**d**) Water cut stage of 95%. White arrows represent the flooding direction.

For the non-conversion schemes (Schemes 1, 2 and 3), the injected HPC system from the injection well mainly plugged along the high-permeability channel, while the equilibrium displacement of the remaining oil occurred in the unswept areas, driving the remaining oil towards the high-permeability channel and production wells. In the subsequent water flooding stage, under the synergistic effect of the residual polymers and the injected HPC system, the flushing of injected water along the high-permeability channel was prevented; in addition, the flow resistance of the injected water from the new wells was increased, making it difficult to break through into the high-permeability channel. More subsequently injected water flowed to the new production wells and displaced the remaining oil in the original unswept areas, further expanding the sweeping range of the water flooding. It can be seen that, compared with Scheme 1 (Figure 18), the new injection well in Scheme 2 (Figure 19) was closer to the high-permeability channel, and the injected water was more likely to break through the HPC system and enter the high-permeability channel, forming a new main displacement path; that is, more injected water from the new injection well entered the high permeability channel and flowed to the original production well. A large amount of the HPC system injected from the new injection well remained at the edge of the model. Although the model was completely swept, its oil displacement efficiency was obviously low. In addition, the new production well was closer to the high-permeability channel, with a smaller displacement pressure difference and a displacement effect that was less balanced than that in Scheme 1.

**Figure 20:**
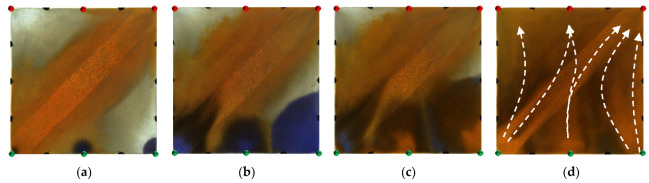
Scheme 3 in WAHPCF experiments. (**a**) Initial stage of HPC injection. (**b**) End stage of HPC injection. (**c**) Initial stage of water injection. (**d**) Water cut stage of 95%. White arrows represent the flooding direction.

**Figure 21:**
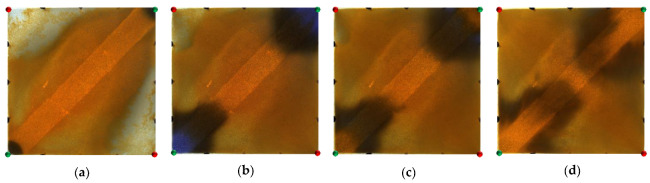
Scheme 4 in WAHPCF experiments. (**a**) Initial stage of HPC injection. (**b**) End stage of HPC injection. (**c**) Initial stage of water injection. (**d**) Water cut stage of 95%.

**Figure 22:**
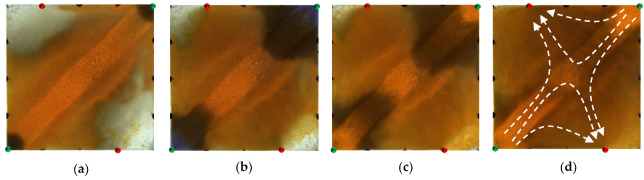
Scheme 5 in WAHPCF experiments. (**a**) Initial stage of HPC injection. (**b**) End stage of HPC injection. (**c**) Initial stage of water injection. (**d**) Water cut stage of 95%. White arrows represent the flooding direction.

In Scheme 3 (Figure 20), after infilling two wells based on Scheme 1, the mainstream direction of the original flow field changed and expanded the action range of the HPC flooding. For the conversion schemes (Schemes 4 and 5), after converting the original production well and infilling two production wells, the mainstream direction of the flow field changed, and the injected HPC system formed an effective plug from both ends of the high-permeability channel, as shown in Figure 21 and Figure 22. Under the synergistic effect of the residual polymer and diversion lines, the subsequently injected water displaced the remaining oil to the production wells in a relatively balanced manner, enlarging the swept area to the entire range of the model. Compared with Scheme 5, a large amount of the HPC system remained near the high-permeability channel, and its plugging effect was not effectively exerted, resulting in unbalanced water flooding. Furthermore, it can be clearly seen that the displacement path of the subsequent water flooding first runs along the high-permeability channel and is then diverted to the production wells at the diversion line.

#### 2.3.2. Quantitative Results Analysis

**Figure 23:**
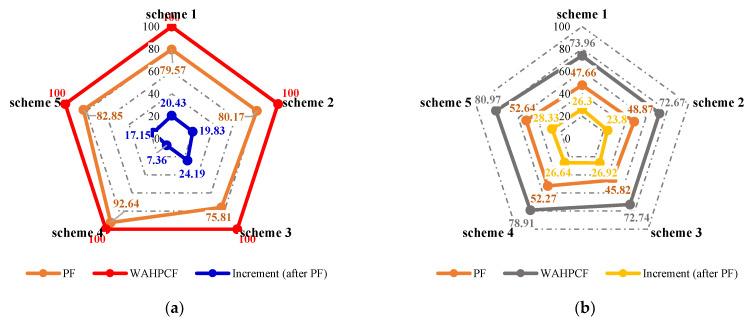
Sweep efficiency and recovery in WAHPCF experiments. (**a**) Sweep efficiency. (**b**) Recovery.

From the sweep efficiency changes at different flooding stages for the five schemes shown in Figure 23a, it can be seen that after polymer flooding, the well adjustment combined with HPC flooding not only significantly utilized the oil in the unswept areas, but also deeply excavated the remaining oil. Compared with the WAF experiments, under the same well pattern adjustment conditions, the sweep efficiencies of the five schemes achieved 100% after HPC flooding, which indicates that the well pattern adjustment had a positive effect on the HPC flooding.

**Figure 24:**
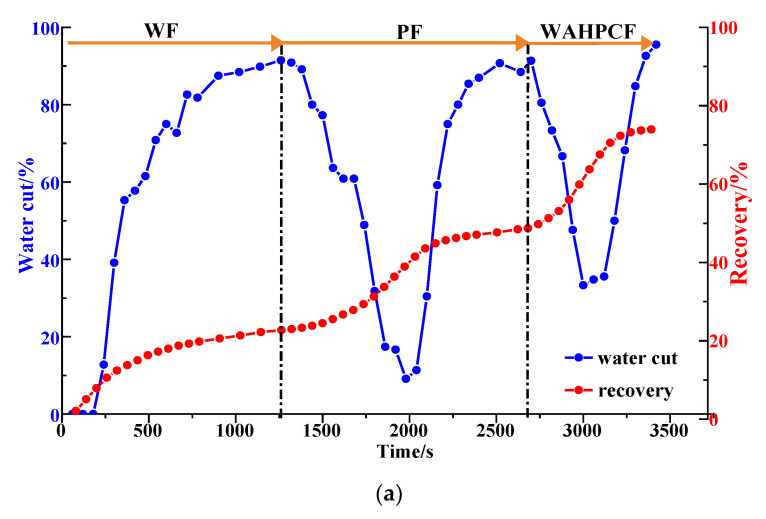

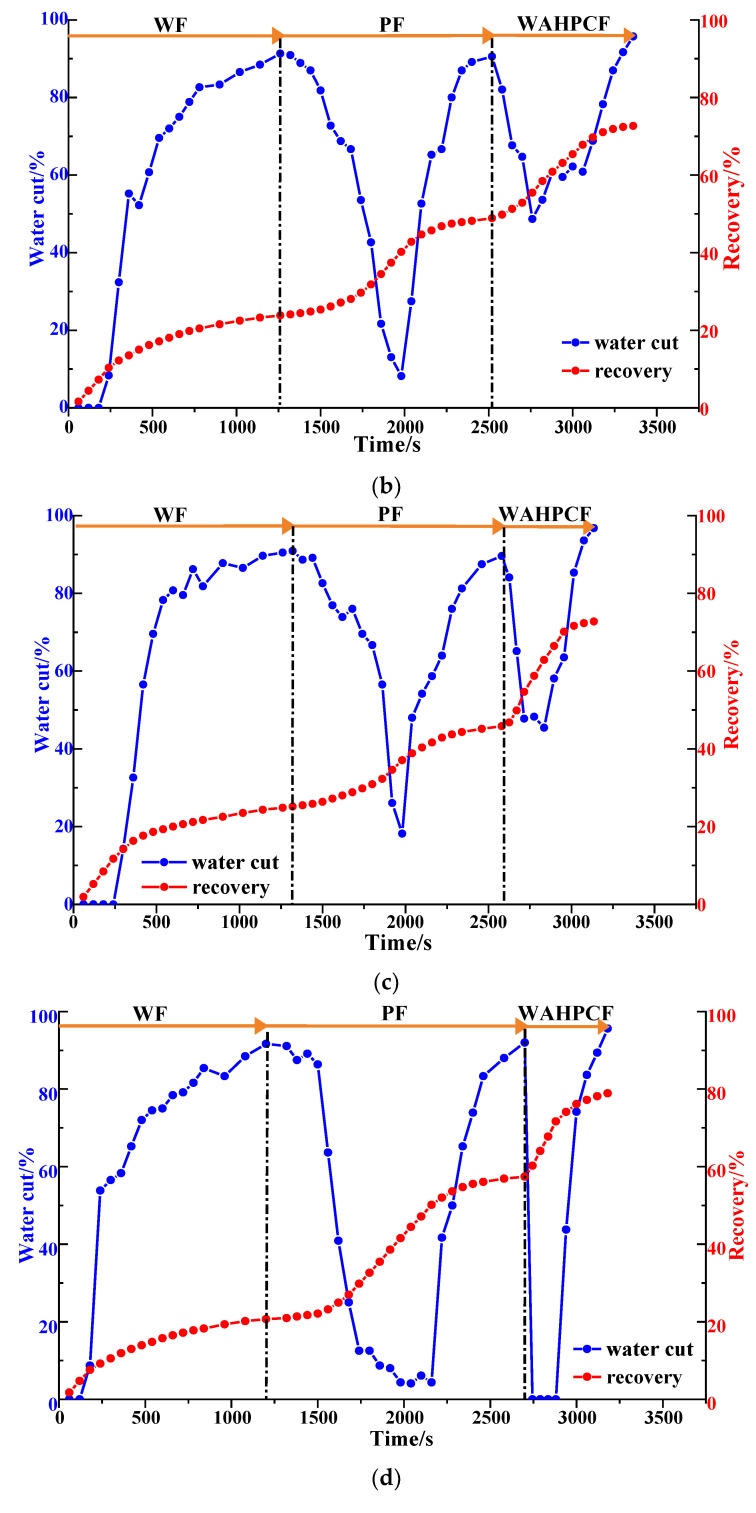

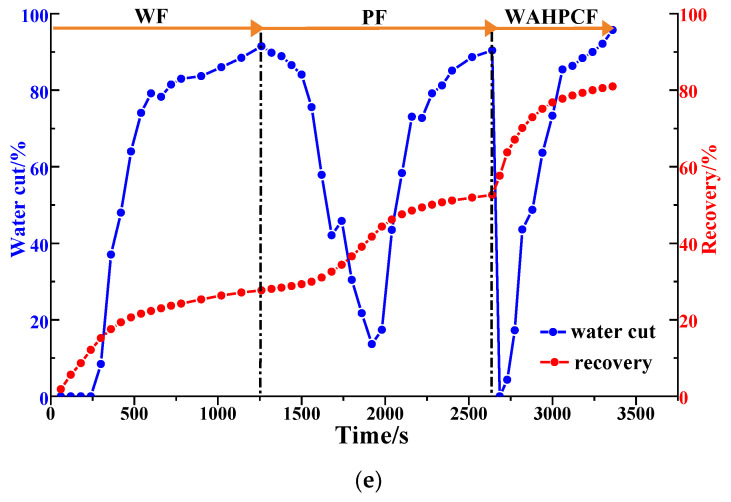
Water cut and recovery change results for five WAHPCF experiments. (**a**) Scheme 1. (**b**) Scheme 2. (**c**) Scheme 3. (**d**) Scheme 4. (**e**) Scheme 5.

The water cut and recovery changes for HPC flooding after different well pattern adjustment schemes are given in Figure 23b and Figure 24. After polymer flooding, the model recovery for each scheme was between 45.82% and 52.64%, and after HPC flooding, it was above 72.67%, with a maximum of 80.97%. Due to the positive effect on HPC flooding following the well pattern adjustments, the water cut reduction in each WAHPCF scheme increased significantly after polymer flooding; the recovery factor also improved greatly, with an increase of more than 23.8 percentage points. Compared with the WAF scheme under the same densification and adjustment conditions, recovery significantly improved. In addition, due to the synergistic effect of multiple chemical agents in the HPC system, the production time for HPC flooding with a water cut lower than 95% was significantly prolonged after well pattern densification and adjustment, as shown in Figure 24 Comparisons between the conversion scheme and the non-conversion scheme show that after well pattern densification and adjustment, the conversion scheme was better than the non-conversion scheme in enhancing oil recovery. Among the schemes, Scheme 5 had the highest EOR value at 28.33%; this was followed by Scheme 3 with an EOR value of 26.92%, and Scheme 2 with the lowest EOR value at 23.8%.

## 3. Conclusions

According to the characteristics of the HPC flooding pilot test block in the Shengli Oilfield, we focused on the characteristics of highly developed dominant channels after polymer flooding, and conducted a series of visualization experiments of well pattern densification and adjustment, HPC flooding and their synergistic regulation, using a two-dimensional sand pack visual displacement device. The main conclusions are as follows:

1. For the reservoirs after polymer flooding, HPC flooding can block the mainstream channel, and significantly increase oil production; however, the injected HPC system mainly advanced along the high-permeability channel between the injection and production wells, and there was still a large area of unswept remaining oil at the edge of the model, with a limited sweep expansion;
2. Well pattern densification and adjustment have a positive effect on HPC flooding, which can divert the original mainstream direction after polymer flooding, and effectively expand the sweeping range. After well pattern densification and adjustment, under the synergetic effect of residual polymers and multiple chemical agents in the HPC system, the water cut reduction significantly increased after polymer flooding;
3. Conversion schemes were better than non-conversion schemes in terms of expanding the sweep efficiency and enhancing oil recovery. After converting the original production well to an injection well and infilling new production wells, the mainstream direction of the original flow field was broken. The injected water was displaced along the high-permeability channel and diverted to the unswept area, effectively expanding the sweeping range of injected water.

## 4. Materials and Methods

### 4.1. Experimental Apparatus

**Figure 25:**
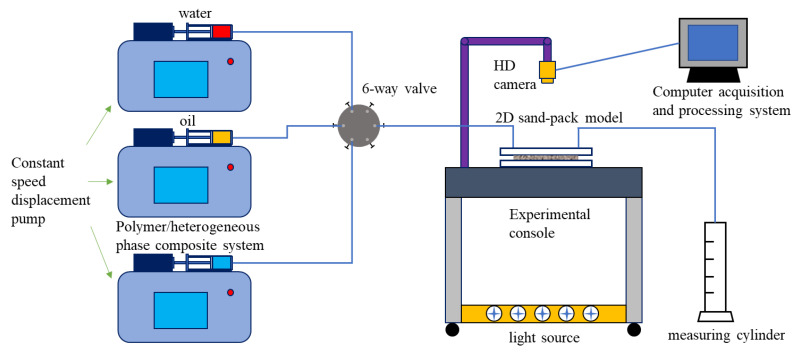
Equipment for visual displacement.

The two-dimensional sand pack visual displacement device that was used is shown in Figure 25. The device comprises a positive displacement pump that can maintain a constant flow and speed, an experimental console, a syringe, an LED light, a high-resolution camera, a measuring cylinder, a computer acquisition and processing system, and a plane sand pack model.

**Figure 26:**
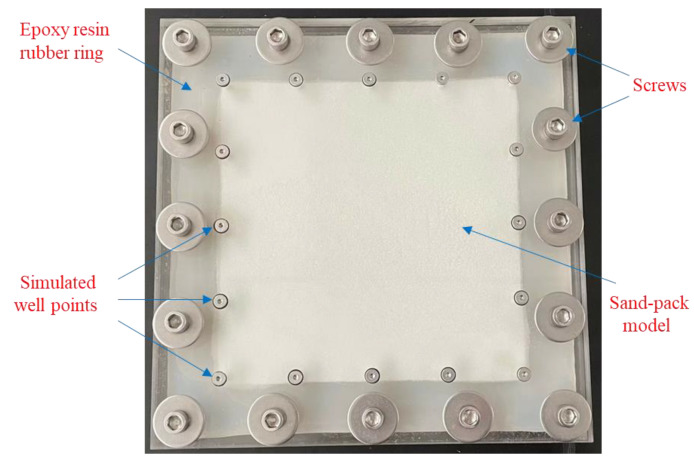
Plane sand pack model.

Figure 26 shows the plane sand pack model, which is composed of two glass plates, one upper and one lower, that are 2 cm thick each. There is a groove in the lower plate where the epoxy resin rubber ring is placed. The overall size of the model is 35 cm × 35 cm, wherein the sand pack area is 28 cm × 28 cm, and 3 mm thick. In addition, the model has 16 screws for compaction sealing; 16 simulated well points are set around the sand pack area.

### 4.2. Experimental Materials

**Figure 27:**
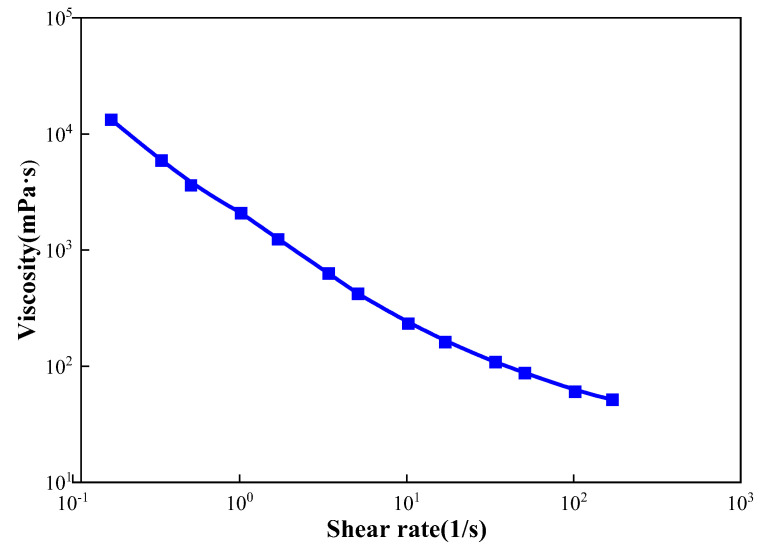
Relationship between apparent viscosity and shear rate of the polymer solution.

Polymer: In this study, the polymer provided by the Shengli Oilfield was partially hydrolyzed polyacrylamide (HPAM) with a molecular weight of 2.0 × 10^7^ and with a relative molecular weight of 89.55% solid content. The concentration of the polymer solution prepared with ultrapure water was 1000 mg/L, and an automated high-pressure and high-temperature viscometer (AMETEK Chandler Engineering, model 5550) was used to test the rheology of the polymer solution at room temperature. As shown in Figure 27, the polymer solution had good rheology at room temperature, and its apparent viscosity decreases with the increase in the shear rate.

**Figure 28:**
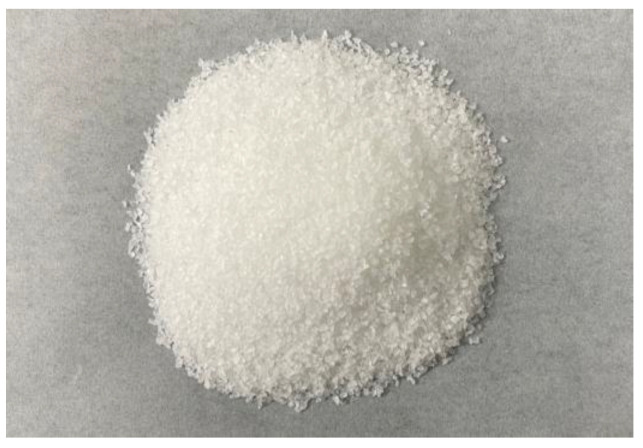
Dry powder B-PPG.

B-PPG: The experimental B-PPG was used in the north area of the Shengli Oilfield; it had an elastic modulus of 10.3 Pa and a particle size of 100–150 mesh. The dry powder B-PPG is shown in Figure 28.

Surfactant: The experimental surfactant provided by the Shengli Oilfield was petroleum sulfonate (molecular formula: C_23_H_38_SO_3_M).

Displacement water: The experimental displacement water was ultrapure water.

Oil: The experiments used a simulated oil configured with industrial lubricating oil and kerosene, with a viscosity of 50 mPa·s and a density of 0.83 g/cm^3^.

**Figure 29:**
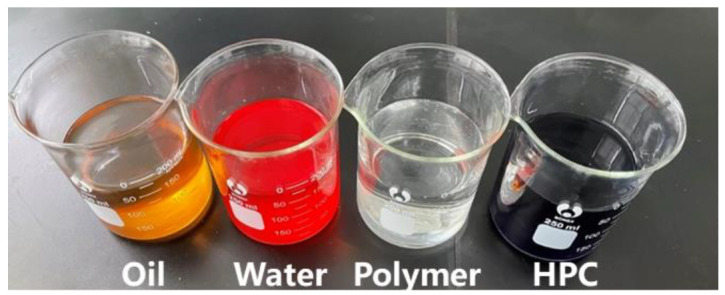
Displacement solution (oil, water, polymer, HPC from left to right).

Dyeing agents: Two dyeing agents, namely methyl orange (molecular formula: C_14_H_14_N_3_NaO_3_S) and methyl blue (molecular formula: C_37_H_27_N_3_Na_2_O_9_S_3_), were used in the experiment to dye the experimental displacement water and HPCF system, as shown in Figure 29.

### 4.3. Experimental Methods

**Figure 30:**
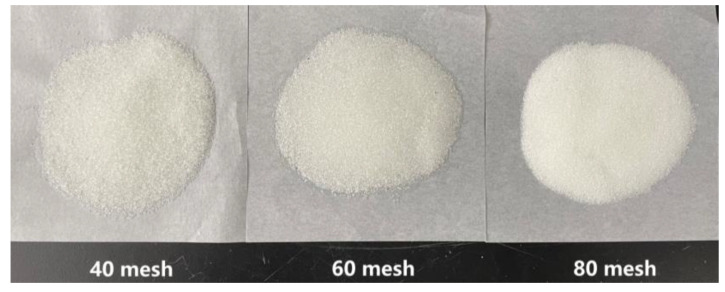
The glass microspheres of different meshes.

The glass microspheres with different meshes that were used for sand-filling were all provided by the same manufacturer. The different meshes of the glass microspheres were used for their different physical properties. The specific sand-filling steps were as follows:

1. Clean the glass microspheres with ultrapure water to remove impurities and dry them at a high temperature (120 °C) to ensure that the wettability of the glass microspheres is consistent [47]. The glass microspheres of different meshes used in the experiments are shown in Figure 30;
2. Pour the dried glass microspheres into the model, and separate and fill the high- and low-permeability zones using a partition. Smooth and compact, and then compress and seal the model with screws;
3. Using the positive displacement pump, inject the ultrapure water into the model at a rate of 3 mL/min, and leave it for 3 h after saturation;
4. Using the positive displacement pump, inject the simulated oil into the model at a rate of 2 mL/min until the outlet oil content reaches 100%, and record the total injected volume of the ultrapure water and simulated oil;
5. Allow the ultrapure water and simulated oil to stabilize in the model.

**Figure 31:**
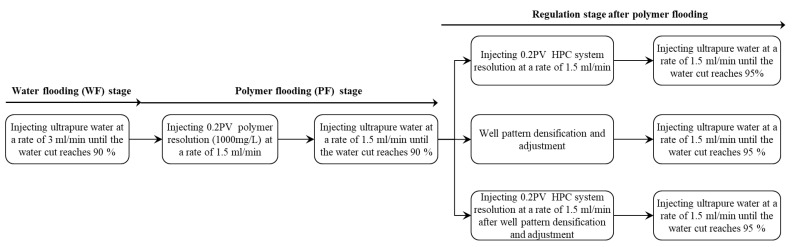
The displacement processes of experimental design.

The experimental displacement process was designed as shown in Figure 31, including the water flooding stage, polymer flooding stage and regulation stage after polymer flooding; the process was conducted at a room temperature of 26 °C. With regard to the regulation stage after polymer flooding, HPC flooding, well pattern intensification and adjustment (WAF), and the combination of the two (WAHPCF) were considered. The polymer, PPG and surfactant concentration of the HPC system were 1000 mg/L, 500 mg/L and 2000 mg/L, respectively, and the well pattern intensification and adjustment were based on the model of the original well pattern unit. During the experiments, some graduated cylinders were used to record the total volumes of fluid and oil produced every 50 s, so as to calculate the water cut and the oil recovery of each flooding stage.

## Figures and Tables

**Table 1:** Oil recovery and sweep efficiency using different processes in heterogeneous models.

Process	Weakly Heterogeneous Model		Strongly Heterogeneous Model	
	Oil Recovery (%)	Sweep Efficiency (%)	Oil Recovery (%)	Sweep Efficiency (%)
Water flooding (WF)	17.50	80.35	22.70	60.31
Polymer flooding (PF)	60.13	92.52	42.02	70.88
HPC Flooding (HPCF)	74.10	98.45	53.45	79.87
Increment (after PF)	13.97	5.93	11.43	8.99

## Data Availability

The data of this article are available from the corresponding author upon reasonable request.

## Abbreviations

WF	Water flooding
PF	Polymer flooding
HPC	Heterogeneous phase composite system
HPCF	Heterogeneous phase composite flooding
WA	Well pattern densification and adjustment
WAF	Well pattern densification and adjustment combined with water flooding
WAHPCF	Well pattern densification and adjustment combined with HPC flooding
